# The social support, mental health, psychiatric symptoms, and functioning of persons with schizophrenia participating in peer co-delivered vocational rehabilitation: a pilot study in Taiwan

**DOI:** 10.1186/s12888-021-03277-0

**Published:** 2021-05-25

**Authors:** Kan-Yuan Cheng, Chia-Feng Yen

**Affiliations:** 1 Department of Psychiatry, Taipei Veterans General Hospital Yuli Branch, Yuli Township, Hualian County Taiwan 98142; 2 Department of Public Health, Tzu-Chi University, Hualian City, Hualian County Taiwan 97071

**Keywords:** Peer support, Mental illness, Vocational rehabilitation

## Abstract

**Background:**

Vocational peer support (VPS) services are recovery-oriented interventions in modern psychiatric care for persons with schizophrenia. However, few VPS services are found in Taiwan. Hence, a pilot program of peer co-delivered vocational rehabilitation to support persons with schizophrenia in Taiwan was proposed and evaluated.

**Methods:**

Six peers were trained and were willing to co-lead and assist workplace problem-solving groups and care skills training in an extended vocational rehabilitation program from August 2017 to December 2018. The social support, mental health, psychiatric symptoms, and functioning of service users were assessed before and after peer co-delivered services, and the assessments were based on the following: Social Support Scale (SSS), Chinese Health Questionnaire-12 (CHQ-12), Brief Psychiatric Rating Scale (BPRS), Global Assessment of Function (GAF), and the Chinese version of the Social Functioning Scale (C-SFS).

**Results:**

The recruited 46 service users were mostly middle-aged (49.1 ± 9.8), with 27 being male (58.7%). After interventions, 42 service users who completed the program had a significantly increased SSS score (149.1 ± 31.8 vs. 161.2 ± 35.0, *df* = 41, *t* = 2.70, *p* = 0.01) and subscale of friend-peer dimension (44.4 ± 12.0 vs. 53.2 ± 13.2, *df* = 41, *t* = 4.72, *p* < 0.001). The objective (GAF: 69.8 ± 9.8 vs. 72.6 ± 8.8, *df* = 41, *t* = 3.50, *p* = 0.001) and subjective social functional scores (C-SFS: 75.2 ± 8.8 vs. 78.1 ± 9.5, *df* = 41, *t* = 2.59, *p* = 0.01) both significantly increased. The weekly wage elevated significantly (37.5 ± 35.5 vs. 43.6 ± 38.0, *df* = 41, *t* = 2.57, *p* = 0.01) and the BPRS-18 score decreased significantly, too (31.2 ± 6.7 vs. 29.3 ± 5.0, *df* = 41, *t* = − 2.83, *p* = 0.007).

**Conclusions:**

Peer co-delivered vocational rehabilitation services may enhance the social support received by persons with schizophrenia and improve their occupational outcomes. The pilot program proposed can thus be a model for non-Western countries with limited resources allocated by governments to support persons with schizophrenia. Trial registration: ClinicalTrials NCT04767204, retrospectively registered on Feb 23, 2021.

## Background

Persons with schizophrenia may see their social and/or occupational functions deteriorate during the course of their illness [1, 2], due to the psychotic, affective, and/or cognitive symptoms triggered by the illness [2, 3]. These individuals also tend to be ill-equipped to make necessary adjustments when facing discrimination in living environments, because of low self-efficacy and internalized stigma [4, 5].

To overcome these challenges, recovery-oriented services have been integrated into mental health care systems for decades to support persons with schizophrenia [6]. Peer-delivered intervention is one such service that has emerged in the past 20 years. Unlike trained professionals, peer support workers who also live with schizophrenia can share lived experiences and make decisions together with service users [7]. Hence, self-determination and self-efficacy are emphasized in peer support services to help service users overcome internalized stigma and the sense of powerlessness [4, 6, 8]. Empirical evidence also shows that peer support services can be effective in instilling hope and overcoming the aforementioned difficulties, and can help persons who experience severe mental illness to engage more actively in their journey of recovery [9, 10]. Delivered as part of the case management service, peer support services can also reduce hospitalization rates and psychiatric symptoms [9, 11].

Given the proven effectiveness of supported employment [12, 13], peer support services have also been integrated into vocational rehabilitation systems for persons who experience severe mental illness [14, 15]. During job matching, peer support services delivered as part of supported employment offer individualized assistance, and the preferences of persons with disabilities are always respected [12, 13]. Peer support workers draw from their own lived experiences to help persons who experience severe mental illness overcome internalized stigma and low self-esteem and/or self-efficacy at work [4, 13]. Peer support workers also serve as role models for persons with disabilities, by exemplifying the positive outcomes that are achievable if persons with disabilities tap into their own preferences, potential, and advantages for employment through self-determination [14, 16]. An earlier study found that peer support worker’s “lived experience” motivated persons with disabilities to engage in relationship-building and instilled in them a sense of normalcy, both of which facilitated the forming of mutual support and the pursuit of vocational goals by persons who experience severe mental illness [14].

In Taiwan, mental health services are primarily provided through hospital-based treatment but they have shifted to community care gradually in recent decades. In 2017, a total of 23,404 beds for psychiatric treatment in hospitals were provided for the general population in Taiwan, with a ratio of 99.4/100,000 persons [17]. This coverage ratio is higher than those in countries where community care for persons with severe mental illness is emphasized (UK: 23.9/100,000, USA: 29.8/100,000 and AUS: 29.0/100,000 between 2015 and 2017) [18]. In contrast to hospital treatment, in 2017, there were 67 community rehabilitation centers and 144 halfway houses in local communities, with ratios of 13.5/100,000 and 25.8/100,000 persons in Taiwan, respectively [17]. Sheltered or supported employment in Taiwan is still primarily delivered by trained professionals or paraprofessionals in community facilities for persons with severe mental illness. Although trained peers can deliver vocational support based on recovery concepts at least as well as professionals in supported employment services [14–16], the large absence of peer support workers is rooted in the lack of insurance coverage and limited government budgets in Taiwan. Accordingly, further investigations are warranted to evaluate the effectiveness of peer support services in vocational rehabilitation among persons with severe mental illness under the development of peer-deliver services in Taiwan.

Therefore, we designed a 2-phase pilot program: Phase I includes the training of peers to become service providers of peer support services through the completion of a curriculum designed by the professionals in community rehabilitation center, and phase II includes the measurement of effectiveness among service users under peer co-delivered services (Table 1). The investigated effectiveness among persons with schizophrenia in this study included the received social support, mental health, psychiatric symptoms, and functional and occupational outcomes.

**Table 1:** Study phases: training & service content, participants, and stakeholders

Phase	Process	Participant	Stakeholder
Phase I: Training for peer support workers	1. Organizing 2 focus group sessions to discuss and decide on the curriculum content	• 8 individuals currently participating in supported employment	• 1 psychiatrist • 2 psychologists • 1 psychiatric nurse • 1 social worker • 1 case manager • 1 occupational therapist
	2. The curriculum includes 12 h each of lecture &practice (2 h in total of lecture & practice per week, for 12 weeks)		
Phase II: Extended vocational rehabilitation co-led & assisted by peer support workers	1. Assessing, by using questionnaires, the social support received by service users, their mental health, psychiatric symptoms and functioning, as well as collecting data of earned (weekly) income and working hours by service users before the intervention	• 6 of 8 peer support workers who completed training and decided to proceed to Phase II of the program • 46 service users currently participating in sheltered or supported employment	• 2 occupational therapists
	2. Peer support workers as coleaders to train service users for workplace problem-solving (1.5 h every 2 weeks, 8 sessions in total)		
	3. Peer support workers as assistants in care skills training (2 h per week, for 16 weeks)		
Post-intervention follow-up	1. Assessing, by using questionnaires, the social support received by service users, their mental health, psychiatric symptoms and functioning, as well as collecting data of earned (weekly) income and working hours by service users after the intervention	• 42 service users completed the extended vocational rehabilitation program	• None
		*Withdrawals:* • 1 service user getting improved skills to care her elderly mother with disability at home • 1 service user suffering acute psychosis • 2 service users failing to meet the threshold of attendance rate of 80%	

## Methods

### Participants

The pilot program was held from April 2017 to December 2018 at the Taipei Veterans General Hospital Yuli Branch (TVGH-YL). The hospital provides treatment and community care for patients with mental illness who reside in the rural area of eastern Taiwan. The TVGH-YL administers a half-way house, a community rehabilitation center, and a supported housing program. The hospital also provides sheltered and supported employment as part of its community care services for persons with mental illness [19]. In the month before the Phase I program (Table 1), we invited participants with schizophrenia who had cared for elderly persons, such as provided meal-delivery, housekeeping or care attendant services under the supported or sheltered employment, to join the training curriculum in the Phase I program, because these persons were peer support worker candidates who could share their work experiences from the Phase II program. The invitation to Phase I was given by the principal investigator through individual interviews and based on the list of recommendations by the staff in the community rehabilitation center. In Phase II (Table 1), we recruited the participants by posting posters on bulletin boards in the half-way house and community rehabilitation center 1 month before initiation.

The inclusion criteria for both phases of the program were: (i) Having disability certification with diagnoses of schizophrenia in the social welfare system, or catastrophic illness of schizophrenia in the health insurance system; (ii) living in half-way houses or receiving services from the supported housing program; (iii) participating in a sheltered or supported employment program; and (iv) showing interest in the job of care attendant. The exclusion criteria were: (i) Comorbidity of severe physical illnesses which could lead to hospitalization; (ii) acute exacerbation of psychosis; and (iii) a reading ability below the age of 6 years. For Phase I, there was one additional inclusion criterion which required experience of caring for elderly persons in the community for at least 1 year.

### Pilot program

#### Training for peer support workers

Initially, there were seven professionals who had at least 5 years of experience in community facilities discussing the framework and content of the training curriculum for peer support workers in the focus groups (Table 1). These seven professionals who are specialized in 6 medical disciplines were also the teachers in the curriculum. Table 2 details the themes of the curriculum with the principles of recovery and operations of peer support. The various intervention levels oriented the teacher to emphasize the range of social connections in the provided services (Table 2). The trainees participated in the curriculum which includes 1–2 classes per week on average, lasting for a total of 16 weeks. Each trainee can be helped by his or her tutor, who is also one of the teachers in the curriculum. At the end of each lecture session, four questions were proposed by the teacher to examine the trainees with respect to the core concepts of the class. In the practice session, the performance of trainees was examined through case discussions, role playing, or simulated situations. There was no final examination in the Phase I program. However, the trainee was required to pass all classes with the assent of the teachers. If the trainee needed to improve their knowledge or skills, he or she was allotted personal time with the teacher to receive more help. Each trainee could only miss two classes and he or she would need extracurricular learning, taught by the tutor.

**Table 2:** Levels of intervention and themes of curriculum for peer support worker training

Intervention level	Themes of curriculum				
	We need peer support	Be healthier and happier	My recovery journey	Recover together	Work together
Community	Introducing the People with Disabilities Rights Protection Act in Taiwan^a^	Using resources in communities to cultivate independent living skills, better health, and to find a job^c^			
	Dealing with discrimination encountered in communities^a^				
Service system	Connecting with other people^b^	Mental health &mental health promotion^a^	Concepts of recovery & empowerment at both individual &service team levels^a^		Coleading workplace problem-solving group^b^
		Treatment & rehabilitation in mental health services^a^	Roles of trained professionals with different specialties &peer support workers in mental health services^a^		Assisting the vocational rehabilitation training^b^
Individual	What is the job of peer support workers? ^a^	Cooperating with care-givers and/or trained professionals with different specialties^b^	Principle of self-determination^a^	Skills of active listening^a^	Leadership in a group^a^
			My life story^a^	Recognizing critical time points^b^	Sharing the decisions made during my own vocational journey^a^
			My experience of managing my own mental illness^b^	Illustrating my recovery story through role-playing^c^	Acquiring interviewing skills through simulations^c^

^a^one-hour lecture session

^b^one-hour practice session

^c^two-hour practice session

#### The extended vocational rehabilitation services co-led & assisted by peer support workers

The Phase II program was held twice, in August of 2017 and June of 2018 (Table 1). Based on the needs of elderly persons with disabilities or dementia in local communities, the 2 occupational therapists organized the extended work training course, which focused on improving the care skills of Phase II program participants (service users). The 2 occupational therapists also held workplace problem-solving groups once every 2 weeks for service users. The above interventions were originally part of supported employment services in the community rehabilitation center, but the participation rate was previously unsatisfactory. Therefore, we integrated the peer support service into this service system. Before each training session, peer support workers discussed with 2 occupational therapists (“stakeholders”) to decide on the content and process of the session. Peer support workers’ involvement should account for at least 50% of the session time to ensure the intensity of support. The supervisor (KYC) had weekly discussions with the occupational therapist and monthly discussions with the peer support worker based on feedback from satisfaction questionnaires for service users and the group records. Four instances of observation by the supervision were also arranged to audit the performance of the occupational therapist and peer support workers in all the sessions of the Phase II program. More specifics on this phase of the program can be found in Table 1.

### Measurements

#### Social support

We used the Social Support Scale (SSS)—initially designed by Liu and later modified by Sung and Yeh [20]—to measure the social support received by service users. The Kaiser–Meyer–Olkin (KMO) value of 0.82 and the Bartlett test of sphericity (BT) of 815.37 (*P* < 0.001) in factor analysis confirm the construct validity of the scale. Internal consistency is also confirmed by the Cronbach’s α of 0.86 [18]. Three dimensions of social support were measured: relatives or family (SSS-R), staff or professionals (SSS-S), and friends or peers (SSS-F).

#### Mental health and psychiatric symptoms

The Chinese Health Questionnaire-12 (CHQ-12) was employed to measure the mental health of service users. The value of the area under the Relative Operating Characteristic curve is 0.85, and the cutoff value is 3/4 [21]. The sensitivity of the questionnaire is 78%, and the specificity, 77% [20]. The lower the score, the better the mental health.

We also measured the psychiatric symptoms of the service users by using the Brief Psychiatric Rating Scale-18 (BPRS-18). According to Bell et al., the α values, which measure internal consistency for positive, negative, and general symptoms in the BPRS, are 0.69, 0.68, and 0.46, respectively, which are deemed satisfactory to acceptable [22]. The interrater reliability (*r* = 0.87) is also deemed satisfactory [22].

#### Social function

We measured the social functions of service users objectively by using the Global Assessment of Functioning (GAF) and subjectively by using the Chinese version of the Social Functioning Scale (C-SFS). Jones et al. reported a reliability coefficient of 0.72 and a significant negative association between the GAF score and patients’ medication/support needs [23].

The Chinese version of the Social Functioning Scale (C-SFS) was adapted by Song [24] from the Social Functioning Scale (SFS) developed by Birchwood et al., by factoring in distinct cultural characteristics in Taiwan. The internal consistencies are deemed acceptable to good, with a Cronbach’s α value of 0.86 for the scale as a whole and Cronbach’s α values ranging from 0.48 to 0.88 for the subscales [24]. The higher the score, the better the social function.

#### Earned income from employment

The weekly wages of service users from sheltered or supported employment were collected from users’ records on file for the 3 months before and after the intervention of vocational rehabilitation. The average weekly income and working hours during the 3 months were used as the measure of occupational outcome.

#### Process of assessment

The pre- and post-intervention self-report questionnaires were completed with assistance from the occupational therapist (MPK). The BPRS-18 and GAF scales were measured by a board psychiatrist (KYC) who had experience in the multiple-center clinical trial. The assistant and investigator both belonged to the intervention group, because there was no control group or blind procedure in the study design.

### Statistical methods

As all indicators examined in this study were continuous variables, we used the paired *t*-test to compare the measurements before and after the intervention. IBM SPSS Statistics 16.0 was employed for statistical analysis.

## Results

### Characteristics of service users

Among the 187 persons with severe mental illness under community care in the study site, 8 peers were trained to be peer support workers during phase I of the program. All the 8 peers passed the training curriculum (Table 2). However, only six persons were willing to provide the services.

Respectively, 24 and 22 service users participated in the Phase II program in the first and second year. Table 3 presents the characteristics of users of the peer-supported vocational rehabilitation services. Most of them are middle-aged, and approximately three-fifths are male. Most are single and have received formal education for more than 9 years. Nearly three-fourths live in halfway houses, while the rest joined the supported housing program. The most common social welfare support which service users have received is disability welfare, followed by exemption from the copayment of health insurance, and low-income subsidy, in that order (Table 3). Roughly two-thirds of service users participated in the sheltered employment program, and the rest joined the supported employment program. The main types of jobs of the service users are cleaner (*n* = 11, 23.9%) and manpower dispatch (*n* = 9, 19.6%) (Table 3). Most service users experienced the onset of mental illness during young adulthood and have a long history of hospitalization. About two-fifths have a history of suicide attempts or violent behavior.

**Table 3:** Characteristics of service users receiving vocational peer support services

Characteristics	*N* = 46	
Demography	n	(%)
Age (mean ± SD)	49.1	± 9.8
Sex		
Male	27	(58.7)
Female	19	(41.3)
Years of education		
6 years	3	(6.5)
7–9 years	10	(21.7)
10–12 years	13	(28.3)
> 12 years	20	(43.5)
Marital status		
Single	44	(95.7)
Married	2	(4.3)
Housing		
Halfway house	34	(73.9)
Supported housing program	12	(26.1)
Social welfare		
Disability welfare	35	(76.1)
Exemption from copayment of health insurance	25	(54.3)
Low-income subsidy	14	(30.4)
Monthly income^a^		
< 100 USD	24	(52.2)
100–199 USD	7	(15.2)
200–299 USD	6	(13.0)
300–399 USD	4	(8.7)
> 400 USD	5	(10.9)
Kinds of job		
Cleaner	11	23.9
Manpower dispatch	9	19.6
Porter	6	13.0
Care attendant	4	8.7
Assistant	4	8.7
Cooker	4	8.7
Others^b^	8	17.4
Physical comorbidity	30	(65.2)
Psychiatric history		
Age of onset (mean ± SD)	25.3	±8.8
Years of hospitalization (median (25–75%til))	8.0	(4.0–13.3)
History of violence or suicide attempt	19	(41.3)

*SD* Standardized deviation

^a^Wages earned from sheltered or supported employment

^b^ Clerk (*n* = 2), creative worker (*n* = 2), sales (*n* = 2) and sheltered employment under training (*n* = 2)

Out of the 46 users who signed up for the vocational rehabilitation support, 4 later withdrew. One experienced acute exacerbation of psychosis and also exhibited the most severe psychiatric symptoms (BPRS-18 = 45) among all service users and poorest self-reported mental health (CHQ = 6) when enrolling in the program. One user gained knowledge and skills caring for her disabled elderly mother at home. The other two failed to reach the attendance rate threshold of 80% (Table 1).

### Pre- vs. post-intervention of peer co-delivered services

As shown in Table 4, despite scoring the lowest before intervention, the social support from friends or peers (SSS-F) is the only dimension that scores significantly higher after the intervention (44.4 ± 12.0 vs. 53.2 ± 13.2, df = 41, t = 4.72, *p* < 0.001), and contributes directly to the higher post-intervention score of social support as a whole (149.1 ± 31.8 vs. 161.2 ± 35.0, df = 41, t = 2.70, *p* = 0.01). The post-intervention score of psychiatric symptoms (BPRS-18) is significantly lower than the pre-intervention score (31.2 ± 6.7 vs. 29.3 ± 5.0, df = 41, t = − 2.83, *p* = 0.007), although the scores of mental health (CHQ-12) do not vary significantly before and after the intervention (3.1 ± 2.6 vs. 2.8 ± 2.5, df = 41, t = − 1.08, *p* = 0.29). The scores of social function as measured by the GAF (69.8 ± 9.8 vs. 72.6 ± 8.8, df = 41, t = 3.50, *p* = 0.001) and the C-SFS (75.2 ± 8.8 vs. 78.1 ± 9.5, df = 41, t = 2.59, *p* = 0.01) increase significantly after the intervention. The weekly wages earned from sheltered or supported employment also increased significantly after the rehabilitation program (37.5 ± 35.5 vs. 43.6 ± 38.0, df = 41, t = 2.57, *p* = 0.01). Weekly working hours did not significantly change after the intervention (20.0 ± 7.6 vs. 21.9 ± 8.7, df = 41, t = 1.72, *p* = 0.09). The increase of income among the service users primarily came from two participants with supported employment originally obtaining competitive employment of care attendant in daycare center and eight shifting the sheltered work to supported employment, housekeeping for the elderly persons.

**Table 4:** Pre- vs. post-interventions: social support, mental health, psychiatric symptoms, social function, and earned income

	Pre-intervention		Post-intervention		*df*	*t*	*P* value
	Mean	SD	Mean	SD			
SSS	149.1	31.8	161.2	35.0	41	2.70	0.01^*^
SSS-R	49.2	14.4	51.0	14.3	41	1.02	0.32
SSS-S	55.5	13.0	57.8	13.9	41	1.63	0.11
SSS-F	44.4	12.0	53.2	13.2	41	4.72	< 0.001^*^
CHQ-12	3.1	2.6	2.8	2.5	41	−1.08	0.29
BPRS-18	31.2	6.7	29.3	5.0	41	−2.83	0.007^*^
GAF	69.8	9.8	72.6	8.8	41	3.50	0.001^*^
C-SFS	75.2	8.8	78.1	9.5	41	2.59	0.01^*^
Weekly working hours	20.0	7.6	21.9	8.7	41	1.72	0.09
Weekly Income (USD)	37.5	35.5	43.6	38.0	41	2.57	0.01^*^

*Statistically significant, *p* < 0.05

*SD* Standard deviation, *SSS* Social Support Scale, *SSS-R* Social Support Scale-Relatives or family, *SSS-S* Social Support Scale-Staffs or professionals, *SSS-F* Social Support Scale-Friends or peers, *CHQ-12* Chinese Health Questionnaire-12, *BPRS-18* Brief Psychiatric Rating Scale-18, *GAF* Global Assessment of Function, *C-SFS* Chinese version of Social Function Scale

## Discussion

In this pilot study, the trained peers could cooperate with therapists in the extended work training course to enhance the social and occupational functions of service users with schizophrenia through the integration of peer support services into the current community settings for mental health in Taiwan. The correlation between social support and occupation has been reported in previous studies [25, 26]. In Switzerland, Rüesch et al. found that subjects (261 persons with schizophrenia or affective disorders) with an occupation tended to have a larger social network and that social support mediated the relationship between occupation and quality of life. However, income was barely or even negatively related to subjects’ perceived quality of life [26]. Rollins and colleagues also reported that workplace network characteristics positively correlated with job satisfaction, after studying 100 persons with severe mental illnesses in Chicago [25]. Yet, workplace network characteristics were not strongly related to hourly wages or the overall job tenure, indicating declining perceived social network support (from supervisors and coworkers) with job tenure [25].

Contrariwise, a significant improvement in weekly wage was found in the current study after persons with schizophrenia received vocational rehabilitation support co-delivered by peers (Table 4). Two factors may have contributed to these divergent findings. First, the pilot program proposed in this study emphasized support from peers who also suffer from mental illnesses themselves, which further enriched the quality of the support and connection between peer support workers and service users. Studies by Rüesch et al. and Rollins et al. examined, instead, the more common social network support at the workplace (from supervisors and coworkers who were not mental illness patients) [25, 26]. Secondly, most of the persons with schizophrenia in our study were middle-aged, had an earlier onset of mental illness, and had a long history of hospitalization. As various dimensions of social support dwindled over time, the peer co-delivered service support proved particularly timely to bridge the growing gap in their need for support. These two factors might also explain the findings in this study showing that the social support from friends or peers (SSS-F) was the only dimension that scored significantly higher after the intervention, despite scoring the lowest among all 3 social support dimensions before the intervention (Table 4).

It is worth noting that the weekly wage earned by the 42 service users in the pilot program grew by 16.7% after the intervention. This increase contrasted with a growth of merely 5% (43.3 USD to 45.5 USD) among the 156 persons with mental illness participating in the supported employment program from 2017 to 2018 at the community rehabilitation center in Yuli. Hence, the peer co-delivered rehabilitation intervention proposed in this study appears to improve the occupational function of persons with mental illness more than the regular rehabilitation program.

Our study also revealed positive effects on social functions of persons with schizophrenia after they received peer co-delivered interventions, as measured both subjectively (C-SFS) and objectively (GAF) (Table 4). This finding echoes the evidence documented in the literature that peer support services can yield more improvement in social functions among individuals with mental illnesses than traditional mental health care. This positive outcome may be derived from the more robust social engagement of service users with peer support workers through mutual interactions and as peer support workers shared their own recovery stories [27]. In our study, as social function also measures the occupational dimension, a higher score associated with the employment component can also lift up the overall social function score. Additionally, the care and skills training that service users received from peer support workers contributed positively to service users’ social functions.

Improvements in psychiatric symptoms and reduced rehospitalization have been reported in studies of persons with mental illness who received peer-delivered services as part of case management [11, 28]. Our study also demonstrated diminished psychiatric symptoms among persons with schizophrenia after peer co-delivered interventions (Table 4). This positive outcome may be underscored by the multi-pronged emphasis placed on mental illness management throughout the vocational rehabilitation sessions. To ensure that service users are ready to care for the elderly with disabilities or dementia (one of the objectives of vocational rehabilitation in this pilot program), the learning of coping skills for psychotic symptoms was elaborated on during the workplace problem-solving sessions and reinforced through role-playing under demonstration by peers. Peer support workers also served as “role models” for service users by sharing experiences of how they managed their own illnesses. Peer support workers’ empathic and nonjudgmental attitudes were conducive to establishing equal relationships with service users, which allowed peer support workers to share their creativity and knowledge with service users and address challenges arising from mental illnesses [8, 27].

Although there was no significant change to mental health in the service users after peer co-delivered intervention (Table 4), the participant with the worst mental health among the users suffered from acute exacerbation of psychosis during the service period. As the vocational rehabilitation program helped service users acquire new care skills, the program also added stress to service users by requiring them to juggle between their current employment and the extended vocational training. The peers or therapists could monitor how service users who are suffering more from mental illness are adjusting during the service period to prevent from acute psychosis under the stress.

There are a number of limitations in this study. First, there was no control group or blind procedure, so possible confounding factors were not excluded, such as the improvement of the vocational rehabilitation system as a whole in the society and changes of wage in the overall labor market during the study period. Second, whether the benefits observed during the current study can be sustained over a longer period of time remains unknown, given the lack of long-term follow-up. The positive effects demonstrated could be the result of focused attention paid during the relatively short timeframe, or the design and implementation of the program by participating professionals trained in various medical disciplines.

## Conclusions

Peer co-delivered services, when integrated into an extended vocational rehabilitation program, can enhance the social support received by persons with schizophrenia and improve their occupational function. The pilot program proposed in the current study can be a model for non-Western countries where only limited resources are allocated from the government to support persons with schizophrenia. The pilot program can also serve as the basis for building a more advanced vocational rehabilitation system jointly supported by peer support workers and trained professionals. The results of this pilot study should be confirmed by further randomized controlled trials to further understand the benefits and risks of peer co-delivered services in the settings of supported employment, especially occupational outcomes and changes in mental illness courses.

## Data Availability

The data used and/or analyzed in this study are available upon request from the first author.

## Abbreviations

SSS: Social Support Scale
CHQ-12: Chinese Health Questionnaire-12
BPRS-18: Brief Psychiatric Rating Scale-18
GAF: Global Assessment of Function
C-SFS: Chinese version of the Social Functioning Scale
